# The Dimensionality of Proposed DSM-5 PTSD Symptoms in Trauma-Exposed Young Children

**DOI:** 10.1007/s10802-019-00561-2

**Published:** 2019-06-06

**Authors:** Anna McKinnon, Michael S. Scheeringa, Richard Meiser-Stedman, Peter Watson, Alexandra De Young, Tim Dalgleish

**Affiliations:** 1 Centre for Emotional Health, Department of Psychology, Macquarie University, North Ryde, New South Wales 2109 Australia; 2 Department of Psychiatry and Behavioral Sciences, Tulane University School of Medicine, 1440 Canal St., MS 8448, New Orleans, 70112 USA; 3 Department of Clinical Psychology, Elizabeth Fry Building, University of East Anglia, Norwich Research Park, Norwich, NR4 7TJ UK; 4 Medical Research Council Cognition and Brain Sciences Unit, 15 Chaucer Rd, Cambridge, CB2 7EF UK; 5 Centre for Children’s Burns & Trauma Research, Centre for Children’s Health Research, University of Queensland, Level 7, 62 Graham Street, South Brisbane, Queensland 4101 Australia; 6 Cambridgeshire and Peterborough NHS Foundation Trust, Cambridge, UK

**Keywords:** DSM-5, Factor analysis, Young children, Posttraumatic stress disorder

## Abstract

A subtype of the posttraumatic stress disorder diagnosis for children 6 years and younger (PTSD-6Y) was introduced in the Diagnostic and Statistical Manual, Fifth Edition (DSM-5). This study utilized confirmatory factor analytic techniques to evaluate the proposed DSM-5 PTSD-6Y factor structure and criterion and convergent validity against competing models. Data for *N* = 284 (3–6 years) trauma-exposed young children living in New Orleans were recruited following a range of traumas, including medical emergencies, exposure to Hurricane Katrina and repeated exposure to domestic violence. The model was compared to DSM-IV, a 4-factor ‘dysphoria’ model that groups symptoms also associated with anxiety and depression, and alternate 1- and 2- factor models. Convergent validity was established against the Child Behavior Checklist (CBCL). Criterion related validity was established by comparing each model to a categorical rating of impairment. The Dysphoria and PTSD-6Y models offered the better accounts of symptom structure, although neither satisfied minimum requirements for a good fitting model. These two models also only showed small levels of convergence with CBCL dimensions. The 1-factor model offered the most compelling balance of sensitivity and specificity, with the 2-factor model and the Dysphoria model following closely behind. These CFA results do not support the symptom clusters proposed within the DSM-5 for PTSD-6Y. Although a 4-factor Dysphoria model offers a better overall account of clustering patterns (relative to alternate models), alongside acceptable sensitivity and specificity for detecting clinical impairment, it also falls short of being an adequate model in this younger age group.

**Electronic supplementary material:**

The online version of this article (10.1007/s10802-019-00561-2) contains supplementary material, which is available to authorized users.

## Introduction

The introduction of posttraumatic stress disorder for children 6 years and younger (PTSD-6Y) in the Diagnostic and Statistical Manual, Fifth Edition (DSM-5 2013) is an important acknowledgement that stress responses of young children show developmental differences compared to adults. Studies leading up to the DSM-5 revealed that PTSD was underdiagnosed in young children (Scheeringa et al. 2001, 2005). Under DSM-IV, to receive a PTSD diagnosis, a young child must have experienced a Criterion A trauma eliciting high levels of affect, presented with at least one re-experiencing symptom, three avoidance symptoms, two arousal symptoms, and shown impaired functioning. This algorithm was problematic as several symptoms from the DSM-IV avoidance cluster (e.g., a sense of hopelessness) were rarely detectable in young children, leading to under-diagnosis of the disorder (Scheeringa et al. 2001, 2005) with consequent effects for funding, and care provision. A proposal for more developmentally sensitive criteria modified the diagnostic algorithm to four symptoms only, removed the requirement for peri-event emotions, and suggested important developmental adaptations. Prior to DSM-5, several studies showed that this alternative algorithm (PTSD-AA) was superior to the DSM-IV algorithm in its alignment with the presence of PTSD-related clinical impairment (De Young et al. 2011; Meiser-Stedman et al. 2008; Scheeringa et al. 2003a, b).

Based on these findings, PTSD-6Y was established as a distinct diagnostic subtype in the DSM-5, mirroring the structure of PTSD-AA. Consequently, the key differences between PTSD with DSM-IV and PTSD-6Y within DSM-5 are the adaptation of symptoms for young children, the addition of a mood item (DSM-5 PTSD-6Y C3), and the requirement of only four symptoms instead of five for diagnosis. However, PTSD-6Y retains the distinctive symptom clustering of the adult diagnosis reflecting an as yet untested assumption that the latent structure of preschool PTSD symptoms mirrors that in adults. Accordingly, symptoms of PTSD-6Y are arranged into re-experiencing, arousal, and avoidance and negative mood and cognition clusters (Table 1: Model 1). The avoidance and negative alterations in cognitions cluster is further subdivided conceptually into two avoidance symptoms (DSM-5 PTSD-6Y C1-C2) and four negative alterations in cognitions symptoms (DSM-5 PTSD-6Y C3–C7), but these are not separate clusters in the diagnostic algorithm. To receive a diagnosis, a child must present with at least four symptoms from the three clusters, alongside functional impairment.

**Table 1:** Confirmatory factor analytic studies examining the structure of Posttraumatic Stress Disorder (PTSD) symptoms in children and adolescents

Study	Sample	Age	Measure	Models tested^a^	CFI^b^	RMSEA^c^
Sack et al. (1997)	*N* = 209 Trauma exposed	12–17	Diagnostic interview	**DSM-5**	0.92	0.03
Anthony et al. (1999)	*N* = 5664	9–19	Self-report	Single factor 2-factor: Re-experiencing +avoidance, arousal+numbing	0.82 0.87	0.10 0.09
				2-factor: Re-experiencing +arousal, avoidance+numbing	0.83	0.10
	Trauma exposed			DSM-IV	0.83	0.10
				**3-factor: Re-experiencing + avoidance, numbing, arousal**	0.91	.07
				Re-experiencing+ numbing+avoidance, fear+anxiety, sleep+concentration problems	0.90	.10
Bal and Jensen (2007)	*N* = 293 Trauma exposed	8–15	Diagnostic interview	**DSM-IV**	0.96	0.04
Giannopoulou et al. (2006)	*N* = 2031 Trauma exposed	9–17	Self-report	*Avoidance, re-experiencing+arousal*	0.97	0.05
				**DSM-IV**	0.96	0.05
				Avoidance, re-experiencing+arousal (orthogonal)	0.92	0.09
				DSM-IV (orthogonal)^5^	0.91	0.09
Saul et al. (2008)	*N* = 4023 Community	12–17	Diagnostic interview	*DSM-IV*	0.94	0.04
				**DSM-5**	0.92	0.03
				*3-factor: Re-experiencing +avoidance, numbing, arousal*	0.94	0.04
Ford et al. (2009)	*N* = 4023 Community	12–17	Diagnostic interview	*DSM-IV*	0.98	0.03
				**2-factor: Re-experiencing, Avoidance + arousal**	0.98	0.03
				*DSM-5*	0.98	0.02
Ayer et al. (2011)^d^	*N* = 1527 Trauma exposed	12–17	Diagnostic interview	*Single factor*	0.98	0.03
				*2-factor: Re-experiencing/arousal, avoidance*	0.99	0.03
				*DSM-IV* *DSM-5* *Dysphoria* *DSM-IV (hierarchical)* *DSM-5 (hierarchical)* *Dysphoria (hierarchical)*	0.98 0.99 0.99 0.98 0.99 0.99	0.03 0.02 0.02 0.03 0.02 0.02
Ayer et al. (2011)^d^	*N* = 1119 Community	12–17	Diagnostic interview	*Single factor*	0.95	0.04
				*2-factor: Re-experiencing+arousal, avoidance*	0.96	0.04
				*DSM-IV* *DSM-5* *Dysphoria* *DSM-IV (hierarchical)* *DSM-5 (hierarchical)* *Dysphoria (hierarchical)*	0.96 0.97 0.97 0.97 0.96 0.97	0.04 0.03 0.03 0.04 0.03 0.03
Kassam-Adams, Marsac, and Cirilli (Sample 1)	*N* = 479 Trauma exposed	8–17	Self-report	*Single Factor* *DSM-IV* **DSM-5** **Dysphoria** *DSM-IV (hierarchical)*	0.90 0.93 0.95 0.94 0.95	0.04 0.04 0.03 0.04 0.03
				2-factor: Re-experiencing, avoidance+arousal	0.91	0.05
Kassam-adams (Kassam-Adams et al.) (Sample 2)	*N* = 204 Trauma exposed	8–17	Diagnostic interview	Single factor *DSM-IV* **DSM-5** **Dysphoria** *DSM-IV (hierarchical)* *2-factor: Re-experiencing, avoidance+arousal*	0.89 0.92 0.96 0.95 0.95 0.93	0.05 0.05 0.04 0.04 0.04 0.05
Chen (2012)	*N* = 268 Trauma Exposed	8–18 yrs	Self-report	Single factor	0.93	0.10
				**2-factor: Re-experiencing + arousal, avoidance**	0.98	0.05
				*DSM-IV*	0.97	0.05
				*2-factor: Re-experiencing+arousal, avoidance (orthogonal)* ^5^	0.94	0.07
				DSM-IV (orthogonal) ^5^	0.84	0.12
Boyes et al. (2012)	*N* = 1025 Community	10–19	Self-report	DSM-IV	0.87	0.07
Wang et al. (2013)	*N* = 1198 Trauma Exposed	11–18	Self-report	*DSM-5* *Dysphoria Model* **5-factor: Dysphoric arousal**	0.96 0.97 0.98	0.07 0.06 0.06
Sumner et al. (2014)	*N* = 2000 Community	12–17	Diagnostic interview	*Dysphoria* *DSM-5* **5-factor: Dysphoric arousal**	1.0 1.0 1.0	0.02 0.02 0.02
Wang et al. (2015)	*N* = 743 Trauma Exposed	11–17	Self-report	*DSM-5* *Dysphoria* *Dysphoric arousal* *Externalizing behaviours* *Anhedonia* **7-factor: Hybrid**	0.92 0.93 0.93 0.94 0.94 0.95	0.05 0.05 0.04 0.04 0.04 0.04
Liu et al. (2016)	*N* = 559 Community	12–18	Self-report	*DSM-5* *DSM-5 Dysphoria* *Dysphoric arousal* *Externalizing* *Anhedonia* **7-factor: Hybrid**	0.95 0.94 0.95 0.95 0.96 0.97	0.04 0.05 0.04 0.04 0.04 0.04

^a^Models italicised either had a good or excellent fit of the data according to the χ2, in addition to the CFI and RMSEA fit indices. Models in bold are the best fitting models

^b^Comparative Fit Index (CFI): The ratio of the difference between the χ2 for the fitted model and the null model divided by the χ2 for the null model with ≥ 0.90 = good fit and ≥ 0.95 = excellent fit. CFI’s meeting either criteria are marked by an asterisk

^c^Root Mean Square Error of Approximation (RMSEA): The amount of unexplained variance left by the models with ≤0.05 suggesting an excellent fit and ≤ 0.08 suggesting a good fit. CFI’s meeting either criteria are marked by an asterisk

^d^This study does not conclude that there is a best fitting model

^e^Orthogonal = an orthogonal model assumes latent factors are independent (not correlated)

DSM-IV (3-factor): DSM-5 B1-B5; C1-C2 + D1-D7; E1-E6

DSM-5 (4-factor): DSM-5 B1-B5; C1-C2; D1-D7; E1-E6

Dysphoria model: DSM-IV B1-B5; C1-C2; c2-D7 + E1-E3; E4-E5

DSM-5 Dysphoria model: DSM-5 B1-B5; C1-C2; D1-D7 + E1-E2; E3-E4; E5-E6

Dysphoric arousal (5 factors): DSM-5 B1-B5; C1-C2; D1-D7; E1-E2; E3-E4; E5-E6

Hybrid (7-factor): DSM-5 B1-B5; C1-C2; D1-D4; D5-D7; E1-E2; E3-E4; E5-E6

Anhedonia (7-factor): DSM-5 B1-B5; C1-C2; D1-D4; D5-D7; E1-E2; E3-E4; E5-E6

We are aware of no empirical examination to date of the factor structure of PTSD symptoms in young children. Establishing the factor structure of PTSD in young children may be helpful for determining whether PTSD has a distinct symptom profile in this age group. One plausible reason the profile could be different is the marked developmental shifts in cognitive and emotional functioning seen in young children, leading to a differential expression of PTSD symptoms relative to older children or adults (Feldman and Vengrober 2011; Scheeringa 2008; Salmon and Bryant 2002). Establishing the PTSD symptom structure in younger children would therefore assist with establishing a developmentally sensitive conceptualization of traumatic stress responses across the lifespan (Scheeringa et al. 2011). If the DSM-5 clustering algorithm for young children lacks validity then this goal would be compromised, and understanding of the etiology and maintenance of PTSD may be impeded. There may be underlying cognitive or biological mechanisms that relate to clusters not currently conceptualised in the DSM, preventing identification of these relationships, and compromising subsequent treatment models targeting these underlying processes. Clinically, alternative factor models will potentially give rise to different PTSD prevalence rates and it may be that clusters not currently outlined in PTSD-6Y have a stronger relationship to clinical impairment.

Confirmatory analytic investigations of the structure of PTSD symptoms have been carried out in sixteen samples of older children and adolescents (See Table 1). A summary of model structures tested in this study is provided in Table 2. We chose to conceptualize PTSD-6Y as a four-factor model as the symptoms are organized across 4 clusters within the DSM-5, even though a PTSD-6Y diagnosis requires 4+ symptoms from only 3 of those clusters. Overall, this four-factor model, consistent with the DSM-5 PTSD-6Y^1^ structure (Table 2; Model 1), was the best fitting model in four samples of older youth (Sack et al. 1997; Saul et al. 2008) and an adequate model in other studies (Ayer et al. 2011; Ford et al. 2009; Liu et al. 2016; Wang et al. 2010, 2015). A unifactorial model (Table 2; Model 2) was adequate in four studies (Anthony et al. 1999; Ayer et al. 2011; Chen et al. 2012; Kassam-Adams et al. 2010), but unsupported in two others (Anthony et al. 1999). A two-factor model (Table 2; Model 3) was the best fitting model in one study (Ford et al. 2009) and an adequate model in two studies (Ayer et al. 2011; Saul et al. 2008). The DSM-IV 3-factor model (Table 2; Model 4) was the best fitting model in one study (Bal and Jensen 2007) and a good fitting model in 8 others (Ayer et al. 2011; Chen et al. 2012; Ford et al. 2009; Kassam-Adams et al. 2010; Saul et al. 2008; Wang et al. 2013). Finally, an alternative 4-factor solution – the so-called Dysphoria Model (Table 2; Model 5), a more complex model grouping symptoms that have a high degree of co-morbidity with anxiety and depression – has been proposed. This model retains the re-experiencing cluster and contains active avoidance, dysphoric arousal and anxious arousal clusters under the rationale that symptoms overlapping with depression and anxiety will cluster more strongly with one another. This model was the best fitting model in four samples (Boyes et al. 2012; Kassam-Adams et al. 2010; Wang et al. 2010) and adequate in eight studies (Ayer et al. 2011; Elhai et al. 2009; Liu et al. 2016; Sumner et al. 2014; Wang et al. 2010, 2013, 2015). In summary, in older youth there is some indication that 1-, 2-, 3-, and 4-factor models all variously meet thresholds for good fit on key fit indices, although for the most part, more complex models fit the data better.

**Table 2:** Specifications of alternative models of posttraumatic stress disorder

Symptoms	Model 1 4-factor DSM-5 PTSD-6Y		Model 2 1-Factor		Model 3 2-factor		Model 4 3-factor DSM-IV		Model 5 4-factor Dysphoria	
B1. Intrusive memories	Re-ex	1+ Sx	PTSD	4+ Sx	Re-ex	1+ Sx	Re-ex	1+ Sx	Re-ex	1+ Sx
B2. Nightmares	Re-ex		PTSD		Re-ex		Re-ex		Re-ex	
B3. Flashbacks	Re-ex		PTSD		Re-ex		Re-ex		Re-ex	
B4. Emotional Reactivity	Re-ex		PTSD		Re-ex		Re-ex		Re-ex	
B5. Physiological reactivity	Re-ex		PTSD		Re-ex		Re-ex		Re-ex	
C1. Thought avoidance	Act-AV	1+ Sx	PTSD		Av-Ar	3+ Sx	Av	1+ Sx	Act-Av	1+ Sx
C2. Avoidance of reminders	Act-AV		PTSD		Av-Ar		Av		Act-Av	
C3. Emotional state	NC&M		PTSD		Av-Ar		Av		Dys-Ar	2+ Sx
C4. Loss of interest	NC&M		PTSD		Av-Ar		Av		Dys-Ar	
C5. Feeling detached	NC&M		PTSD		Av-Ar		Av		Dys-Ar	
C6. Reduction in positive affect	NC&M		PTSD		Av-Ar		Av		Dys-Ar	
D1. Sleeping difficulties	Ar	2+ Sx	PTSD		Av-Ar		Ar	2+ Sx	Dys-Ar	
D2. Irritability	Ar		PTSD		Av-Ar		Ar		Dys-Ar	
D3. Concentration	Ar		PTSD		Av-Ar		Ar		Dys-Ar	
D4. hyper-vigilance	Ar		PTSD		Av-Ar		Ar		Anx-Ar	
D5. Exaggerated startle	Ar		PTSD		Av-Ar		Ar		Anx-Ar	

*PTSD* PTSD cluster/model, *Av-Ar* Avoidance-Arousal cluster, *Anx-Ar* Anxious Arousal cluster, *Dys-Ar* Dysphoric-Arousal cluster, *Act-Av* Active Avoidance cluster, *AV* Avoidance, *AR* arousal, *NC&M* Negative Cognitions and Mood, *Re = Ex* Re-Experiencing

Given the questions this raises for projecting adult structural models onto youth samples, the primary aim of the present study was to explore the latent structure of DSM-5 post-traumatic stress symptoms in young children aged 3–6 years using confirmatory factor analysis. The DSM-5 PTSD-6Y 4-factor model (Table 2; Model 1) was compared to the aforementioned competing models tested in the adult and older child literatures: a cluster free 1-factor account (Table 2; Model 2 – the basis for DSM-5 acute stress disorder), a 2-factor solution [arousal/avoidance and re-experiencing] (Table 2; Model 3), the DSM-IV 3-factor model (Table 2; Model 4) and the alternative 4-factor Dysphoria model (Table 2; Model 5). Based on the literature in older age children and adolescents, which showed that in the majority of studies more complex models fit the data better than more parsimonious models, we tentatively predicted that a similar pattern might be observed in our young child data. We did not make a prediction as to whether PTSD-6Y or the Dysphoria model would be superior as both have performed similarly well in studies of older age children and adolescents.

The main aim of any DSM diagnosis is to identify a child that is in clinical need, and therefore a second goal was to establish the clinical validity of each model. To do this, we investigated the convergent validity of the different diagnostic models with the exception of DSM-IV, which has shown to be poor in numerous previous studies, to internalizing and externalizing subscales of the Child Behavior Checklist (CBCL). We chose to look at the relation of a general ‘internalizing’ dimension as it has been shown to differentiate youths with and without anxiety (Seligman et al. 2004) as well as PTSD (Haller and Chassin 2012) symptoms in adolescents. PTSD is thought to be associated with both issues of externalization (e.g., anger and irritability) and internalization (e.g., thought avoidance). Based on previous research carried out in older age children and adolescents (Haller and Chassin 2012), it was predicted that PTSD would be associated with both internalizing and externalizing dimensions, but would show the stronger association with the internalizing dimension. We also investigated the criterion validity of the different models in relation to the presence of clinical impairment, conceptualized as being impaired in at least one area of functioning (e.g., relationships with parents, teachers etc.) (cf., Kassam-Adams et al. 2012).

## Method

### Participants

Data on 284 trauma-exposed children (Mean age = 5.10 years, SD = 1.08, 62% male) recruited in New Orleans were examined (Scheeringa et al. 2012). Children had experienced a variety of traumas, including Hurricane Katrina (*n* = 137; 76%), medical injuries (e.g., road traffic collisions) (*n* = 62; 22%), and/or repeated traumas such as domestic violence (*n* = 85; 30%).

Inclusion criteria were that the child: (i) experienced at least one life-threatening trauma when they were old enough to remember it with a narrative recall (typically at least 3 years old) and (ii) was aged between 36 and 83 months at the time of the most recent trauma. Exclusion criteria were: (i) a Glasgow Coma Scale score of <7 in the emergency room; (ii) intellectual disability; (iii) autism spectrum disorder; (iv) blindness; (v) deafness; and (vi) foreign language-speaking families.

Details of recruitment and sample characteristics are described elsewhere (Scheeringa et al. 2012). The study was approved by the Tulane University Committee on Use of Human Subjects, and written informed consent was obtained from the primary caregiver.

A summary of the core characteristics of the sample are presented in Table 3. All reporters were the primary maternal caregiver by design. This was the mother in most cases, but there were a few cases in which grandmothers or aunts were the primary caregiver. No fathers were reporters in the study. The majority of children were of African American (66.5%) descent, followed by Caucasian (21.5%), Mixed race (8.5%) and Other Racial Denominations (3.5%). Roughly half of the mothers taking part in the study (45.8%) were employed. Mothers and fathers tended to be in their mid-thirties at the time of the assessment and both had roughly 12 years of education. Approximately 23% of fathers lived in the family home.

**Table 3:** Demographics of the trauma exposed sample

M (SD)	Single *N* = 62	Multiple events *N* = 85	Hurricane *N* = 137	Total *N* = 284
Age	5.2 (1.1)	5.1 (1.1)	5.1 (1.0)	5.1 (1.1)
Male gender % (n)	68% (*n* = 42)	65% (*n* = 55)	57% (*n* = 78)	62% (*n* = 175)
Ethnicity % (n)				
African Caucasian Mixed race Other race	82% (*n* = 51) 11% (*n* = 7) 5% (*n* = 3) 2% (*n* = 1)	62% (*n* = 53) 18% (*n* = 15) 15% (*n* = 13) 5% (*n* = 4)	62% (*n* = 85) 29% (*n* = 39) 6% (*n* = 8) 4% (*n* = 5)	67% (*n* = 189) 22% (*n* = 61) 9% (*n* = 24) 4% (*n* = 10)
Age of mother	28.9 (6.5)	31.2 (8.2)	34.5 (10.9)	32.3 (9.6)
Age of father	30.4 (5.4)	33.6 (7.2)	34.1 (8.8)	33.2 (7.8)
Years Education (mother)	12.4 (2.3)	12.0 (2.3)	13.7 (2.5)	12.9 (2.5)
Years Education (father)	11.9 (2.3)	11.7 (1.9)	13.0 (2.7)	12.4 (2.6)
Father lives in home % (n)	23% (*n* = 14)	7% (*n* = 6)	34% (*n* = 46)	23.2 (*n* = 66)
Mother employed % (n)	60% (*n* = 37)	28% (*n* = 24)	50% (*n* = 69)	46% (*n* = 130)

### Measures

The Preschool Age Psychiatric Assessment (PAPA: Egger et al. 2006) is a parent-report semi-structured interview assessing mental health disorders in early childhood according to DSM-IV and ICD-10 descriptions. The PTSD module contained developmental modifications to symptoms for the young age group (in line with the PTSD-AA) ensuring that the list of symptoms measured in this study accurately captures the list of DSM-5 PTSD-6Y symptoms even though data were collected prior to the DSM-5 publication. For each item, the interviewer asked respondents to indicate whether the child exhibited behaviours by asking them to consider whether the child differed from the average child of that age, probing the respondent for examples if a symptom was positively endorsed. Items were rated as either present or absent using a categorical response scale (0 = no, 1 = yes). Previous research suggests the test-retest reliability of the PAPA PTSD module over an 11-day period is acceptable (Kappa = 0.73) (Egger et al. 2006). In a previous study, interrater reliability for the PTSD (Kappa = 0.75) module was also found to be acceptable (Scheeringa et al. 1995). Interrater agreement was not established in the present study because we did not have multiple raters for the same participant’.

#### Impairment

A child was considered to be experiencing impairment if one or more of items from the 6 PAPA impairment domains were endorsed (Egger et al. 2006): relationships with parents, siblings, teachers, peers, inability to act appropriately outside the home, and overall levels of distress. For impairment items, interviewers asked respondents if the behaviors interfered with functioning in each area more than the average child of that age. The items were scored dichotomously as absent or present, with impairment on one or more domains considered to be indicative of the presence of impairment. Rating each item involved judgements of both respondents and interviewers. When a respondent endorsed an impairment domain as being present for the child, the interviewer then asked for an example before deciding whether to rate the item positively.

#### Child Behavior Checklist (CBCL: Achenbach 1991)

Parents completed the CBCL to rate their children’s expression of psychological problems along internalizing and externalizing dimensions. If the caregiver’s child was aged 3 to 5 years, the caregiver completed the 1.5 to 5 years of age version (100 items) of the CBCL. If the caregiver’s child was aged 6 years, the caregiver completed the 6 to 18-year-old version (112 items). The scale has been validated on a host of samples (Seligman et al. 2004). Respondents answer items on a 3-point scale (0 = not true; 2 = very true). The psychometrics of the CBCL have been widely established in a wide range of nationally representative samples and data on the convergent validity of the CBCL has been reported in a previous study (Scheeringa et al. 2012).

### Data Analysis

The CFA was carried out using EQS v6.1 (Bentler 2006). The four models compared against the DSM-5 PTSD-6Y (4-factor) (Table 1: Model 1) were: a cluster-free 1-factor account (Table 1: Model 2), a 2-factor [arousal/avoidance + re-experiencing] (Table 1: Model 3), a 3-factor DSM-IV (Table 1: Model 4) and a 4-factor Dysphoria model (Table 1: Model 5).

Models were tested under two conditions. In the first, the models were specified without controlling for any variables. In the second, we identified demographic and trauma related variables that held univariate associations with PTSD, in this case trauma type (single versus repeated), age, and gender. MIMIC modelling was used to account for these univariate variables. Variables were specified in the relevant step of the regression equation for each item. In each condition, the association covariance matrix for each model was estimated using robust maximum likelihood estimation (Raykov and Marcoulides 2006) as recommended when testing categorical variables in small or intermediate-sized samples that have skewed distributions (Lee et al. 1995).

Multiple fit indices were used based on recommendations that fit indices are influenced by sample size, model parameters, and data-normality (Bentler 2007). Well-established fit indices were tested such as the Comparative Fit Index (CFI), Tucker-Lewis Index (TLI), and the Root Mean Square Error-of-Approximation (RMSEA; 90% confidence interval) (Bentler 2007; Moschopoulos and Canada 1984). There is considerable controversy regarding the appropriate cut-off points fit and the number of fit indices that must be met to infer a good model fit (Schermelleh-Engel et al. 2003). Values of 0.95 on the CFI and TLI (Kline 2005) and RMSEA scores <0.05 (Browne and Cudeck 1992) indicate an excellent fitting model. Values of >0.90 on the CFI and TLI (Kline 2005) and RMSEA scores between 0.05–0.08 indicate a good fit (Browne and Cudeck 1992). Schwarz’s Bayesian Information Criterion (BIC) uses the the Satorra-Bentler scaled Chi-squared goodness-of-fit test (Satorra and Bentler 1994) and the number of model parameters to compare models. A BIC score for a given model that is >10 points lower than the next lowest model provides evidence for the superiority of one model over another (Raftery 1995). According to Hair et al. (2006), the value of factor loadings must reach at least 0.30 to be considered of practical significance.

Criterion-related validity, prevalence rates and convergent validity were examined in SPSS v.21. Whether children met the diagnostic requirements for each factor model, with the exception of meeting the impairment criterion, was calculated. To do this, we needed to derive a PTSD diagnosis based on each model, including those models not published in the DSM. In doing this (summarized in Table 1), we felt it was crucial to retain the 4+ total symptom count (Scheeringa et al. 2003b). The associations between these diagnostic algorithms and categorical impairment was computed to examine the criterion validity of the models. An algorithm with good clinical utility would have high scores on the sensitivity index and a low rate of false positives (high scores on the specificity index). As stated earlier, in deciding between algorithms typically more weight is placed on the sensitivity index in recognition of the costs associated with a missed diagnosis (Kirk 2004).

## Results

### Preliminary Analysis

Rates of symptom endorsement ranged from 13.4% (*n* = 38) for ‘C4 loss of interest’ to 75.5% (*n* = 214) for ‘B4 emotional reactivity’ (See Supplementary Table I for full summary). Furthermore, the majority of items had correlations with total symptoms at or above 0.40, with two items correlating at 0.30 or above (flashbacks, reduction in positive affect), suggesting the model was reliable.

### Factor Structure of the Competing PTSD Models

Table 4 presents the fit indices for each model. The DSM-5 PTSD-6Y and the 4-factor Dysphoria Model were excellent fitting models according to the RMSEA (< 0.05) whereas all other models were good fitting models (RMSEA ≤0.08). None of the models met the minimum requirement for a good fit according to the CFI or TLI (CFI & TLI < 0.90). BIC showed that the two alternative 4-factor models – the Dysphoria Model and PTSD-6Y outperformed the other models, but were indistinguishable from one another (<10 point difference). However, after conducting MIMIC modelling to take account of the impact of covariates on these relationships (Supplementary Table III), none of the models met the minimum requirement of a good fitting model.

**Table 4:** Fit indices for the five PTSD models (*N* = 284)

Item models	BIC^a^	CFI^b^	RMSEA (90% CI)^c^	TLI^d^
**Model 1: DSM-5 PTSD-6Y**	**−396.64**	**0.87**	**0.046; 0.032, 0.059**	**0.85**
Model 2: 1-Factor	−386.64	0.79	0.057; 0.045, 0.069	0.76
Model 3: 2-Factor	−383.44	0.80	0.057; 0.045 0.069	0.76
Model 4: DSM-IV	−372.47	0.79	0.058; 0.046, 0.070	0.75
**Model 5: Dysphoria**	**−401.44**	**0.88**	**0.044; 0.030, 0.057**	**0.86**

^a^Bayesian Information Criterion

^b^Comparative Fit Index

^c^Root Mean Square Error of Approximation

^d^Tucker Lewis Index

Models in bold indicate the best fitting models

Table 5 presents factor loadings for the two stronger models – the PTSD-6Y and the Dysphoria models. The same two items from both models did not met the required threshold (> 0.30), PTSD-6Y B1 intrusive memories and B3 flashbacks. A post hoc analysis investigated the impact of removing these two items leading to the Dysphoria model achieving an acceptable model fit on all three indices (RMSEA<0.05, CFI & TLI > 0.90) and the PTSD-6Y on two of the three indices (RMSEA<0.05, CFI > 0.90, but TLI < 0.90). BIC again did not distinguish the two models (Raftery 1995; < 10 point difference) (Supplementary Table IV). These findings stood when MIMIC modelling was employed to take into account important co-variates of symptom structure.

**Table 5:** Factor loadings for DSM-5 PTSD-6Y and Dysphoria models

	DSM-5 PTSD-6Y				Dysphoria			
DSM-5	Re-Exp	Act-Av	NC&M	Ar	Re-Exp	Act-Avoid	Dys-Arous	Anx-Ar
B1. Intrusive memories	0.29				0.26			
B2. Nightmares	0.37				0.35			
B3. Flashbacks	0.27				0.26			
B4. Reactivity	0.54				0.54			
B5. Physiological reactivity	0.39				0.39			
C1. Thought avoidance		0.65				0.65		
C2. Avoidance of reminders		0.38				0.37		
C3. Emotional state			0.48				0.43	
C4. Loss of interest			0.57				0.49	
C5. Feeling detached			0.44				0.43	
C6. Positive affect			0.33				0.31	
D1. Sleeping				0.44			0.48	
D2. Irritability				0.48			0.48	
D3. Concentration				0.36			0.42	
D4. Hyper-vigilance				0.33				0.34
D5. Startle				0.46				0.42

PTSD-6Y = Posttraumatic stress disorder: preschool subtype. *Anx-Ar* anxious arousal cluster; *Dys-Ar* dysphoric arousal, *Act-Av* Active Avoidance, *AV* Avoidance, *AR* arousal, *NC&M* Negative Cognitions and Mood, *Re-Exp* re-experiencing

### The Convergent and Criterion Validity of the Competing PTSD Model Algorithm

Table 6 presents the correlations of presence/absence of diagnosis according to each of the model algorithms (0 = did not meet diagnosis; 1 = met diagnosis) with the child’s continuous scores on the Internalizing (*M* = 56.57, *SD* = 15.27) and Externalizing CBCL subscales (*M* = 56.23, *SD* = 16.09). All models had small (Cohen 1988) correlations with the Externalizing Subscale, Internalizing Subscale, and the Total scale score (Cohen 1988).

**Table 6:** Pearson correlations between diagnostic algorithms and scores on the child behavior checklist

	CBCL-I	CBCL-E	CBCL-T
Models			
DSM-5 PTSD-6Y ^a^	0.39**	0.27**	0.35**
1-factor^b^	0.39**	0.26**	0.33**
2-factor^c^	0.40**	0.29**	0.35**
4-factor dysphoria^d^	0.42**	0.32**	0.39**

PTSD-6Y = DSM-5 Posttraumatic stress disorder: preschool subtype. *CBCL-E* Child Behaviour Checklist Externalizing Scale, *CBCL-I* Child Behaviour Checklist Internalizing Scale, *CBCL-T* Child Behaviour Checklist Total Scale

^a^1+ symptoms must be endorsed from the re-experiencing, 1+ symptoms from active avoidance and the negative mood and cognition, 2 + symptom from the arousal, and impairment in functioning

^b^4+ symptoms must be endorsed from the list of 16 symptoms and impairment in functioning

^c^3+ symptoms must be endorsed from the avoidance/arousal,1+ symptoms must be endorsed from the re-experiencing, and impairment in functioning

^d^1+ symptoms must be endorsed from each of the re-experiencing, 1+ from active avoidance, 2+ from anxious arousal and dysphoric arousal, and impairment in functioning

The ability of each of the models (without the removal of items post hoc) to categorize impairment was investigated. Strong clinical utility involves high scores on the sensitivity index and a low rate of false positives (high scores on the specificity index). Generally, greater weight is placed on sensitivity, recognizing the costs associated with missing the diagnosis of a child who needs help (Kirk 2004).

One hundred and eighty-four children rated positively for impairment. Table 7 presents the sensitivity, specificity, positive predictive value (PPV), negative predictive value (NPV), percentage of children correctly classified as suffering impairment, and PTSD prevalence rates, based on the ‘diagnosis’ of PTSD across the different models (with the exception of the DSM-IV model). PTSD prevalence rates for all four models were > 30% –slightly higher than typically found in older youth (Hiller et al. 2019). Overall, the 1-factor and 2-factor models provided the better balance of sensitivity (0.79 to 0.72) and specificity (0.42 to 0.56) to detect categorical impairment, correctly classifying 65.8% and 65.5% of cases, respectively. The Dysphoria model followed close behind, outperforming the PTSD-6Y, with a sensitivity of 0.70, specificity of 0.54, and correct classification of 65.1% of cases.

**Table 7:** Performance of different symptom requirements per post-traumatic stress clusters and overall models to predict concurrent ratings of impairment (*N* = 184/284)

	Frequency^e^	Sensitivity	Specificity	PPV	NPV	Correctly classified	Diagnosed
DSM-5 PTSD-6Y^a^	150	0.61	0.62	0.75	0.46	61.3%	39.4%
1-factor^b^	203	0.79	0.42	0.71	0.52	65.8%	51.1%
2-factor	178	0.72	0.54	0.74	0.51	65.5%	46.5%
Avoidance/Arousal model^c^							
4-factor Dysphoria^d^	173	0.70	0.56	0.75	0.50	65.1%	45.4%

PTSD-6Y=Posttraumatic stress disorder: preschool subtype. *NPV* negative predictive value; *PPV* positive predictive value

^a^1+ symptoms must be endorsed from re-experiencing, 1+ symptoms from active avoidance and the negative mood and cognition, and 2 + symptoms from arousal

^b^4+ symptoms must be endorsed from the list of 16 symptoms

^c^3+ symptoms must be endorsed from the avoidance/arousal and 1+ symptoms must be endorsed from re-experiencing

^d^1+ symptoms must be endorsed from the re-experiencing, 1+ symptoms from active avoidance, and 2+ symptoms from anxious arousal and dysphoric arousal

^e^The number of children in the sample endorsing meeting symptom requirements with the exception of meeting the impairment criterion

## Discussion

This study aimed to evaluate for the first time the latent structure of PTSD symptoms in young children, following the introduction of PTSD for children 6 years and younger (PTSD-6Y) in the DSM-5. PTSD-6Y was compared to four alternative models evaluated in previous research. The 4-factor ‘Dysphoria’ and PTSD-6Y models provided the better accounts of symptom structure, although neither model met the requirements of a good fitting model without removal of the poorly loading flashback and intrusion symptom items. The partial support for these two models on fit indices did not remain after MIMIC modelling to control for differences in symptom expression associated with trauma type, gender, and age.

In terms of criterion validity, out of these two models the Dysphoria model offered the better balance of sensitivity and specificity. The more parsimonious 1-factor solution offered the best conceptualization in this respect, but none of the models was particularly strong. In terms of convergent validity, the two 4-factor models also displayed only small levels of convergence with CBCL dimensions. The findings indicate that current models of PTSD symptom clustering derived from the adult field do not do a compelling job of capturing the symptom profiles of trauma-exposed preschoolers.

This failure to find an adequate model fit with all symptoms included is at odds with CFAs in older children where it is common to find multiple excellent fitting models involving the full list of symptoms (Ayer et al. 2011; Elhai et al. 2009; Ford et al. 2009; Kassam-Adams et al. 2010; Saul et al. 2008). Low rates of endorsement do not explain the finding as flashbacks were endorsed by 18.3% of the sample and intrusions were endorsed by nearly half the sample (44.7%). One interpretation is that flashbacks and intrusions are not a central component of the construct in young children, although this is inconsistent with clinical observation. Another possibility is the potentially poor validity of parent report for these symptoms in young children (Meiser-Stedman et al. 2007). A final possibility is that the range of PTSD symptoms enshrined in the DSM for young children is too narrow and detailed qualitative investigations of young children’s clinical presentations are indicated, in line with similar endeavors in adults (e.g., Kendler 2016).

Some aspects of the study merit comment. Despite being a diverse sample, almost half of the children experienced only one trauma type (Hurricane Katrina; 48%) and only 30% experienced an interpersonal stressor. Furthermore, only 23% of the fathers in the sample lived in the family home, which is lower than American prevalence estimates found in the general population and may have had some impact on rates of clinical prevalence. How representative the current sample is of the wider trauma population is therefore an important empirical question for future research. It will also be important to replicate these findings in a clinic-attending sample. Finally, to test criterion validity we used the same impairment criterion required for the diagnosis. It is important to replicate our findings around criterion validity using an impairment criterion that is more distant from the diagnosis; for example, a measure of quality of life.

There are other important avenues for future research. Additional tests are needed to evaluate the predictive validity of competing models and their impact on recovery trajectories and relapse rates. In line with the goals of the Research Domain Criteria (Insel et al. 2010) and other trans-diagnostic developmental work (McLaughlin et al. 2011), a logical progression of the present study involves exploratory factor analytic work across disorders in preschool children including core pathology common to depression, anxiety and adjustment disorders, as well as other core psychological processes associated with early stress responses. Further research is also needed in larger samples to determine the differences in psychopathology expressed by simple and complex trauma groups.

To summarize, the present study suggests that in young children, when considering the latent structure of PTSD symptoms there is inconclusive support for four separable symptom clusters as proposed in the DSM-5. Overall, the results suggest that PTSD factor models largely established in the adult literature do not provide an adequate fit of symptom clustering in pre-school children.

## Electronic supplementary material

ESM 1 (DOC 83 kb)

## Footnotes

1 The DSM-5 4-factor model was derived from a model termed in the lead up to the new manual in the empirical literature as the ‘Emotional Numbing’ model.
