# Supplementary material for: The Dimensionality of Proposed DSM-5 PTSD Symptoms in Trauma-Exposed Young Children
Source: J Abnorm Child Psychol. 2019 Jun 6;47(11):1799–809. doi: 10.1007/s10802-019-00561-2 (PMC6805819; doi:10.1007/s10802-019-00561-2)
Supplement: Supplementary file 1 — (DOC 83 kb) [file 10802_2019_561_MOESM1_ESM.doc]

*Supplementary Table I. Symptom counts and item total correlations for the main list of symptoms.*

| PTSD Symptoms | Symptom Count % (n) | Symptom total correlation |
| --- | --- | --- |
| B1. Intrusive memories | 44.7 (127) | .40** |
| B2. Nightmares | 42.3 (120) | .47** |
| B3. Flashbacks | 18.3 (52) | .34** |
| B4. Emotional Reactivity | 75.4 (214) | .54** |
| B5. Physiological reactivity | 17.3 (49) | .46** |
| C1. Thought avoidance | 28.5 (81) | .54** |
| C2. Avoidance of reminders | 53.9 (153) | .44** |
| C3. Emotional state | 28.9 (82) | .44** |
| C4. Loss of interest | 13.4 (38) | .44** |
| C5. Feeling detached | 13.7 (39) | .39** |
| C6. Reduction in positive affect | 14.8 (42) | .31** |
| D1. Sleeping difficulties | 57.4 (163) | .51** |
| D2. Irritability | 62.3 (177) | .53** |
| D3. Concentration | 35.2 (100) | .45** |
| D4. hyper-vigilance | 26.1 (74) | .41** |
| D5. Exaggerated startle | 37 (105) | .53** |

*Supplementary Table II. Fit Indices for the five PTSD models adjusting for the impact of trauma type, age and gender (N=284).*

| Item Models | | χ2(df)^a^ | *p* | | BIC^b^ | | CFI^c^ | | 90% CI RMSEA^d^ | | TLI^e^ |  |
| --- | --- | --- | --- | --- | --- | --- | --- | --- | --- | --- | --- | --- |
| Model 1: DSM-5 PTSD-6Y | χ2(91)=212.02 | | | <.001 | | -302.04 | | .78 | | .069;  .056  .080 | .67 | |
| Model 2: 1-Factor | χ2(90)=208.32 | | | <.001 | | -300.09 | | .79 | | .068;  .056  .080 | .68 | |
| Model 3: 2-Factor | χ2(88)=206.37 | | | <.001 | | -290.74 | | .79 | | .069;  .057  .081 | .67 | |
| Model 4: DSM-IV | χ2(85)=174.13 | | | <.001 | | -306.03 | | .84 | | .061;  .048  .074 | .74 | |
| Model 5: Dysphoria | χ2(85)=181.40 | | | <.001 | | -298.76 | | .83 | | .063:  .050  .076 | .72 | |

Note

^a^ Satorra Bentler χ^2^; ^b^ Bayesian Information Criterion; ^c^ Comparative Fit Index; ^d^ Root Mean Square Error of Approximation; ^e^ Tucker Lewis Index.

*Supplementary Table III. Fit Indices for the five PTSD models after the removal of poorly fitting items* (N=284).

| Item Models | | χ2(df)^a^ | *p* | | BIC^b^ | | CFI^c^ | | 90% CI RMSEA^d^ | | TLI^e^ | |
| --- | --- | --- | --- | --- | --- | --- | --- | --- | --- | --- | --- | --- |
| Model 1: DSM-5 PTSD-6Y | χ2(71)=104.57 | | | <.001 | | -296.50 | | .92 | | .041;  .022  .057 | .89 |  |
| Model 2: 1-Factor | χ2(77)=146.46 | | | <.001 | | -288.52 | | .83 | | .057;  .042  .070 | .8- |  |
| Model 3: 2-Factor | χ2(76)=144.827 | | | <.001 | | -284.50 | | .83 | | .057;  .042  .070 | .80 |  |
| Model 4: DSM-IV | χ2(74)=144.14 | | | <.001 | | -273.20 | | .83 | | .058;  .044  .072 | .79 |  |
| Model 5: Dysphoria | χ2(71)=92.126 | | | <.001 | | -308.95 | | .95 | | .032:  .004  .050 | .91 |  |

Note

^a^ Satorra Bentler χ^2^; ^b^ Bayesian Information Criterion; ^c^ Comparative Fit Index; ^d^ Root Mean Square Error of Approximation; ^e^Tucker Lewis Index

*Supplementary Table IV. Correlation Matrix (N=284) summarizing the relationship of the CBCL to PTSD factor models.*

|  | CBCL  React | CBCL  Anx/Dep | CBCL  Somatic | CBCL  Withdraw | CBCL  Sleep | CBCL  Attention | CBCL  Aggression | CBCL-I | CBCL-E | CBCL-T | PTSD-6Y | 1-factor | 2-factor | Dysphoria |
| --- | --- | --- | --- | --- | --- | --- | --- | --- | --- | --- | --- | --- | --- | --- |
| CBCL  React | 1 | .81** | .41** | .68** | .53** | .45** | .69** | .84** | .69** | .81** | .28** | .28** | .30** | .29** |
| CBCL  Anx/Dep |  | 1 | .40** | .66** | .59** | .41** | .55** | .81** | .59** | .76** | .34** | .28** | .30** | .32** |
| CBCL  Somatic |  |  | 1 | .44** | .30** | .25** | .26** | .63** | .30** | .51** | .13* | .16** | .15* | .18** |
| CBCL  Withdraw |  |  |  | 1 | .54** | .43** | .57** | .74** | .59** | .74** | .26** | .24** | .24** | .27** |
| CBCL  Sleep |  |  |  |  | 1 | .40** | .43** | .59** | .48** | .67** | .36** | .24** | .30** | .37** |
| CBCL  Attention |  |  |  |  |  | 1 | .60** | .49** | .70** | .67** | .19** | .17** | .19** | .23** |
| CBCL  Aggression |  |  |  |  |  |  | 1 | .61** | .91** | .80** | .24** | .24** | .26** | .26** |
| CBCL-I |  |  |  |  |  |  |  | 1 | .71** | .90** | .39** | .39** | .40** | .42** |
| CBCL-E |  |  |  |  |  |  |  |  | 1 | .90** | .27** | .26** | .29** | .32** |
| CBCL-T |  |  |  |  |  |  |  |  |  | 1 | .35** | .33** | .35** | .39** |
| PTSD-6Y |  |  |  |  |  |  |  |  |  |  | 1 | .67** | .80** | .82** |
| 1-factor |  |  |  |  |  |  |  |  |  |  |  | 1 | .82** | .68** |
| 2-factor |  |  |  |  |  |  |  |  |  |  |  |  | 1 | .80** |
| Dysphoria |  |  |  |  |  |  |  |  |  |  |  |  |  | 1 |

CBCL-I=Child Behaviour Checklist – Internalizing Problems; CBCL-E=Child Behaviour Checklist – Externalizing Problems; CBCL-T=Child Behaviour Checklist – Total Problems
